# TR-57 Treatment of SUM159 Cells Induces Mitochondrial Dysfunction without Affecting Membrane Potential

**DOI:** 10.3390/ijms25021193

**Published:** 2024-01-18

**Authors:** Artem Mishukov, Ekaterina Mndlyan, Alexey V. Berezhnov, Margarita Kobyakova, Yana Lomovskaya, Ekhson Holmuhamedov, Irina Odinokova

**Affiliations:** 1 Center of Theoretical Problems of Physico-Chemical Pharmacology, Russian Academy of Sciences, 109029 Moscow, Russia; artem.mishukov1999@gmail.com; 2 Institute of Theoretical and Experimental Biophysics, Russian Academy of Sciences, 142290 Pushchino, Russia; mndlyaneyu@gmail.com (E.M.); ritaaaaa49@gmail.com (M.K.); yannalomovskaya@gmail.com (Y.L.); ekhson@gmail.com (E.H.); 3 Institute of Cell Biophysics, Russian Academy of Sciences, Federal Research Center “Pushchino Scientific Center for Biological Research of the Russian Academy of Sciences”, 142290 Pushchino, Russia; alexbereg56@gmail.com

**Keywords:** TR-57, SUM159 TNBC cells, respiratory complexes: mitochondrial membrane potential, FoF_1_-ATPase, IF1, calcium-regulated mitochondrial ATP-Mg/Pi carrier

## Abstract

Recent works identified ClpXP, mitochondrial caseinolytic protease, as the only target of imipridones, a new class of antitumor agents. Our study of the mechanism of imipridone derivative TR-57 action in SUM159 human breast cancer cells demonstrated mitochondrial fragmentation, degradation of mitochondrial mtDNA and mitochondrial dysfunction due to inhibition of Complex I and Complex II activity. Complete inhibition of oxidative phosphorylation accompanied 90, 94, 88 and 87% decreases in the content of Complex I, II, III and IV proteins, respectively. The content of the F_O_F_1_-ATPase subunits decreased sharply by approximately 35% after 24 h and remained unchanged up to 72 h of incubation with TR-57. At the same time, a disappearance of the ATPIF1, the natural inhibitor of mitochondrial F_O_F_1_-ATPase, was observed after 24 h exposure to TR-57. ATPase inhibitor oligomycin did not affect the mitochondrial membrane potential in intact SUM159, whereas it caused a 65% decrease in TR-57-treated cells. SUM159 cells incubated with TR57 up to 72 h retained the level of proteins facilitating the ATP transfer across the mitochondrial membranes: VDAC1 expression was not affected, while expression of ANT-1/2 and APC2 increased by 20% and 40%, respectively. Thus, our results suggest that although TR-57 treatment leads to complete inhibition of respiratory chain activity of SUM159 cells, hydrolysis of cytoplasmic ATP by reversal activity of F_O_F_1_-ATPase supports mitochondrial polarization.

## 1. Introduction

Recently, a new class of imipridone-derived antitumor molecules, including maternal TRAIL-inducing compound ONC201/TIC10 [1] and newly synthetized derivatives known as TR-compounds (Madera Therapeutics, Chapel Hill, NC, USA) [2] attracted the attention of investigators. The first data set on the potent efficacy of the first-in-class small molecule ONC201 was published in 2013 [3], and over 10 years, a lot of preclinical work has appeared on tumor cells related to hematological and solid malignancies including prostate, breast, ovarian lung and pancreatic cancer cell lines as well as leukemia, lymphoma, glioma and hepatocellular cancer cells [4]. The broad specificity of the action of imipridones, as well as their high efficiency and selectivity for tumor cells, made it possible to use them in clinical trials [4,5,6]. While the early studies showed that the effect of imipridones on tumor cells occurs through inducing the expression of TRAIL and its receptor DR5 [3,7], as well as increasing the sensitivity of cells to exogenous TRAIL [8], recent observations demonstrated mitochondrial caseinolytic serine protease ClpXP, localized within the mitochondrial matrix as the only intracellular target for imipridones [2,9,10]. In the mitochondrial matrix, ClpP forms a complex with the ATP-dependent protein unfoldase ClpX, which belongs to the AAA+ class (ATPase Associated with various cellular Activities) proteases [11]. The ClpXP complex is involved in mitochondrial quality control; its substrates are many mitochondrial proteins, including proteins of the electron transport chain (ETC), tricarboxylic acid (TCA) cycle, mitochondrial gene transcription and translation and ribosomal proteins [12]. The discovery of imipridones-induced activation of ClpP refocused investigators to study massive remodeling and unregulated proteolysis of proteins in mitochondria, accompanied by dramatic morphological changes and fragmentation of mitochondria, degradation of mitochondrial DNA and a rapid decrease in the level of mitochondrial transcription factor TFAM, responsible for maintaining the number of mtDNA copies [2,9,13,14,15].

In addition, RNA sequencing of MB231 cells treated with ONC201 showed changes in the expression of genes responsible for maintaining the mitochondrial genome and other mitochondrial processes, including oxidative phosphorylation [13]. In the current work, we focused on the mechanism of action of TR-57 (a chemically modified derivative of ONC201) with higher (50–100 times) efficacy compared to ONC201 [2,16,17], which is associated with a higher affinity of their binding to ClpP [2]. Multiple in vitro tests have shown that the antitumor effect of TR-57 is rather cytostatic and inhibits cell proliferation without induction of apoptotic cell death [13,18,19,20], and its cytostatic effect manifested not only by inhibition of tumor cell proliferation in vitro [2,21,22]. Characteristically, most of the studied breast cancer cell lines are characterized by the absence of induction of apoptosis under the influence of ONC201 or TR-57; only blocking of cell growth is observed [6], and with prolonged incubation with imipridones, the cells transform into a senescence-like phenotype [21,23]. However, it is still not completely clear what factors determine the sensitivity of cells to the action of imipridones, and the question of how imipridones-treated tumor cells containing mitochondria with impaired respiratory function maintain their viability remains unexplored.

Here, we demonstrated that the mitochondrial component of the antiproliferative effect of TR-57 in cultured SUM159 human breast cancer cells consists of the degradation of the critical structural and functional proteins of mtDNA translation and transcription, associated with a decline in the expression of the critical proteins of the respiratory Complexes I-IV, leading to suppression of the mitochondrial respiratory chain activity. Surprisingly, loss of the activity of the mitochondrial respiratory chain was not associated with depolarization of remodeled mitochondria, and regardless of fragmentation and inhibition of electron transport, drug-treated cells retained mitochondrial membrane potential. Our data indicate that mitochondrial polarization could be supported by the reversal of the activity of mitochondrial FoF1-ATPase, which, due to the consumption of glycolytic ATP, maintains the mitochondrial membrane potential necessary to perform mitochondrial functions and sustain the viability of tumor cells.

## 2. Results

### 2.1. Effect of TR-57 on Mitochondrial Morphology and the Expression of Key Mitochondrial Regulatory Proteins in Cultured SUM159 Cells

**Figure 1:**
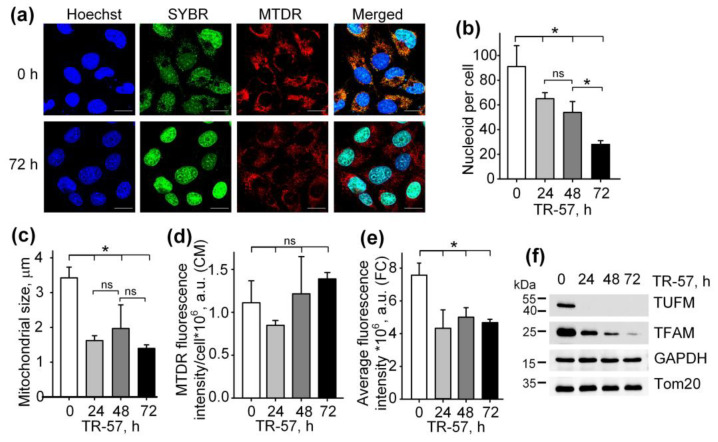
Effect of TR-57 on the number of mitochondrial nucleoids, mitochondrial morphology and size and the expression of key mitochondrial regulatory proteins of SUM159 cells. (**a**) Representative confocal fluorescent images of intact (0 h) and treated with 150 nM TR-57 for 72 h (72 h) SUM159 cells, loaded with Hoechst 33342 (Hoechst, blue), SYBR Green I (SYBR, green) and MitoTracker Deep Red (MTDR, red); Scale bar—20 µm. Number of mitochondrial nucleoids (**b**); average mitochondrial size (**c**) and average intensity of MitoTracker Deep Red fluorescence (**d**) in SUM159 cells treated with 150 nM TR-57 for 0 (intact), 24, 48 and 72 h measured by confocal fluorescent microscopy (CM); (**e**) average intensity of MTDR fluorescence measured by flow cytometry (FC); (**f**) Western blot analysis of mitochondrial proteins TUFM, TFAM and Tom20 in SUM159 cells treated with 150 nM TR-57 for 0, 24, 48 and 72 h. The data are presented as the means ± SD of at least three independent experiments. The data were analyzed using a one-way ANOVA with a post-hoc Bonferroni test. *—*p* < 0.05, ns—*p* > 0.05.

To study the effect of TR-57 on the morphological and functional characteristics of mitochondria in human triple-negative breast cancer cells SUM159, cells were exposed to 150 nM TR-57 for 24, 48 and 72 h and stained with fluorescent dyes specific to mitochondria (Mito Tracker Deep Red 633 (MTDR)) and DNA (SYBR Green I). The concentration of 150 nM TR-57 was chosen according to literature data. Graves and coauthors previously found that for SUM159 cells, the IC_50_ was equal to 14 nM TR-57 [2], but later researchers always used TR-57 in concentration ~10-fold higher than IC_50_ [2,23,24]. Figure 1a and Supplementary Figure S1 show the confocal fluorescent microscopy data where mitochondria/mitochondrial mass are colored red (MTDR), cell nuclei and double-stranded mtDNA is colored green (SYBR Green-I), and cell nuclei alone are colored blue (Hoechst 33342). The data obtained on the confocal microscope were processed using Fiji open source software (https://fiji.sc/ (accessed on 1 January 2021)) [25], and the quantitative results are presented in Figure 1b–d. Incubation of SUM159 cells with TR-57 for 24 h caused a decrease in the content of mitochondrial nucleoids from 91.06 ± 16.97 to 65.04 ± 4.85 nucleoids per cell; further incubation for 48 h and 72 h reduced the number of nucleoids to 53.93 ± 8.80 and 28.01 ± 2.97, respectively (Figure 1b). The decreased number of mtDNA after TR-57 treatment coincided with the decline in the content of TFAM and TUFM, critical proteins involved in the regulation of the stability, transcription and translation of mtDNA, confirmed by Western blot analysis (Figure 1f). The content of mitochondrial elongation factor TUFM decreased to undetectable values after 24 h of incubation with TR-57. In comparison, the level of mtDNA transcription factor TFAM decreases gradually with the duration of the treatment and is found in trace amounts after 72 h (Figure 1f).

The average size of mitochondria stained with MTDR, which was calculated from confocal images, decreased approximately 2-fold after 24-h incubation of cells with TR-57 and did not change significantly with further treatment (Figure 1c) indicating that mitochondrial fragmentation occurs in the first 24 h of incubation with TR57. MitoTracker Deep Red fluorescence intensity per cell, reflecting mitochondrial mass, decreased by 25% after 24 h of incubation with TR-57 and, with further incubation, increased by 25%; however, the spread of values for different cells was quite large, and the changes did not meet the reliability criterion (Figure 1d). Total mitochondrial content was also measured using flow cytometry, which showed that the average fluorescence intensity of MitoTracker Deep Red decreased by 40% over 24 h and remained at this level until 72 h of incubation with TR-57 (Figure 1e).

Thus, we confirmed previously the published data that treatment of breast cancer cells with substances of the imipridone group (ONC201 and/or TR-57) causes a decrease in the content of mitochondrial nucleoids and mitochondrial fragmentation [13,21,26]. We have shown that the decrease in the number of nucleoids occurs gradually and depends on the time of incubation of cells with the agent, while the decrease in the size and number of mitochondria occurs in the first 24 h and is not dramatic.

### 2.2. Functional State of the Respiratory Chain of Mitochondria in SUM159 Cells Treated with TR-57

**Figure 2:**
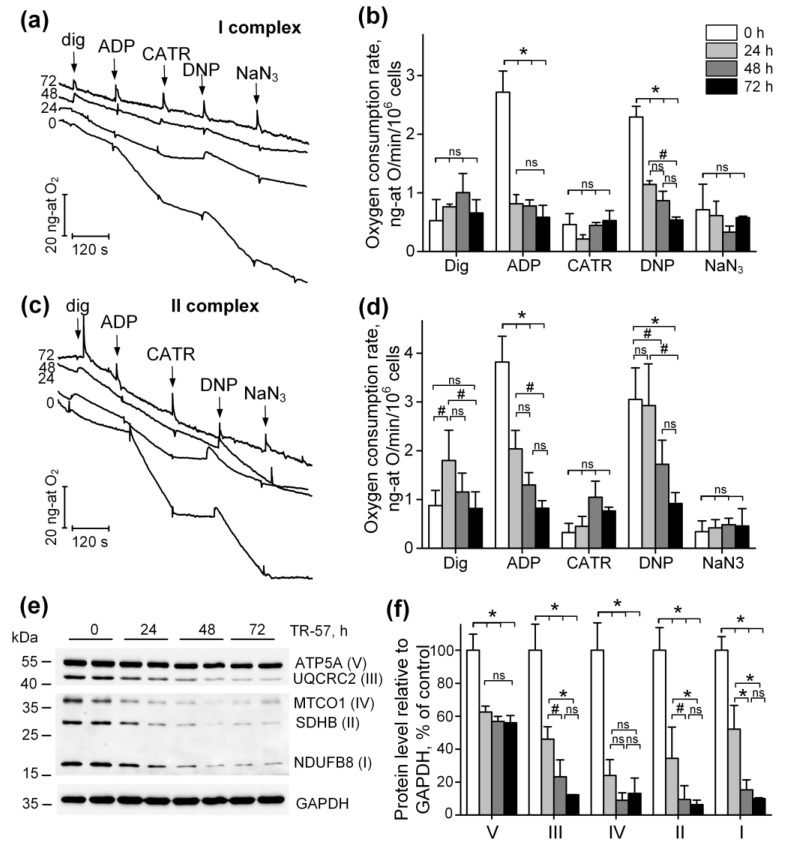
TR-57 gradually decreases mitochondrial respiratory chain activity and electron transport chain (ETC) protein content. (**a**,**b**) Representative curves and oxygen consumption rates SUM159 cells, control (white bars) and treated with 150 nM TR-57 for 24 h (light gray bars), 48 h (dark gray bars) and 72 h (black bars), oxidizing Complex I substrates; (**c**,**d**) representative curves and oxygen consumption rates of SUM159 cells oxidizing Complex II substrates; arrows on curves indicate additions of 0.003% digitonin, 2 mM ADP, 5 μM carboxyatractyloside (CATR), 30 μM 2,4-dinitrophenol (DNP) and 1 mM NaN3; (**e**) representative Western blot of ETC complex proteins; (**f**) quantitative analysis of ETC proteins normalized to GAPDH level. The ratio of protein level to GAPDH under control conditions was taken as 100%. The data are presented as the means ± SD of at least three independent experiments. The data were analyzed using a one-way ANOVA with a post-hoc Bonferroni test. * *p* > 0.001, # *p* > 0.01, “ns”—non-significant.

To characterize the functional state of mitochondria in SUM159 cells treated with TR-57, we assessed mitochondrial oxygen consumption in a model of permeabilized cells in the presence of respiration substrates for Complex I (pyruvate + malate) and Complex II (rotenone + succinate) of the respiratory chain. Figure 2a shows typical recordings from an oxygen electrode, which are characteristic of cellular respiration using respiration substrates of Complex I (Figure 2a). The tracing of oxygen concentration changes within the suspension after 0, 24, 48 and 72 h of treatment of SUM159 cells with TR-57 (150 nM) is shown in Figure 2a. The first addition of 0.003% digitonin to the cell suspension and permeabilization of the plasma membrane (dig) led to the activation of the basal rate of respiration. The addition of 2 mM ADP (ADP) initiated a State 3 respiration (oxidative phosphorylation rate), which was blocked by the addition of 5 μM carboxyatractyloside (CATR), an adenine nucleotide translocator inhibitor (Figure 2a). The maximal (uncoupled) rate of respiration was achieved in the presence of 30 µM 2,4-dinitrophenol (DNP), and non-mitochondrial oxygen consumption was assessed in the presence of the cytochrome oxidase inhibitor NaN_3_ (Figure 2a, NaN_3_). Figure 2b demonstrates the averaged values of respiration rates using Complex I substrates for each time point. These data demonstrate that 24 h of incubation of cells with TR-57 suppresses oxidative phosphorylation (2.72 ± 0.36 ng-atom O/min/10^6^ cells in the control to 0.81 ± 0.16 ng-atom O/min/10^6^ cells) as well as uncoupled respiration (Figure 2b, ADP). Longer incubation of cells with TR-57 decreases the oxygen consumption rate to 0.58 ± 0.20 ng-atom O/min/10^6^ cells. DNP-induced respiration rate decreases gradually from 2.29 ± 0.18 ng-atom O/min/10^6^ cells in the control to 1.14 ± 0.06 ng-atom O/min/10^6^ cells (24 h with TR-57), 0.87 ± 0.16 ng-atom O/min/10^6^ cells after 48 h and 0.54 ± 0.05 ng-atom O/min/10^6^ cells after 72 h of incubation of cells with TR-57 (Figure 2b). Similarly, the respiration of SUM159 cells, oxidizing Complex II substrate, are affected by different duration of exposure to TR-57 as shown in Figure 2c,d, the typical recordings and the averaged values of the rate of respiration by digitonin-permeabilized SUM159 cells, oxidizing Complex II substrates, respectively. Thus, the ADP-dependent and DNP-dependent respiration under these conditions reveals a gradual decrease in respiration rates depending on the time of incubation of cells with the agent. State 3 (phosphorylating), respiration decreased from 3.82 ± 0.53 ng-atom O/min/10^6^ cells in the control to 2.04 ± 0.38 ng-atom O/min/10^6^ cells (24 h of treatment), 1.30 ± 0.25 ng-atom O/min/10^6^ cells after 48 h, and to 0.82 ± 0.15 (72 h of incubation) with TR-57. Similarly, uncoupled respiration decreased from 3.05 ± 0.65 ng-atom O/min/10^6^ cells to 2.93 ± 0.85, 1.72 ± 0.49 and 0.92 ± 0.22 ng-atom O/min/10^6^ cells after 24, 48 and 72 h, respectively. Thus, the long-term incubation of SUM159 with 150 nM TR-57 results in consistent suppression of mitochondrial respiration supported by both Complex I and Complex II substrates.

Observed inhibition of the respiratory activity of mitochondria following long-term exposure to TR-57 complemented by a decline in the expression of the key proteins—subunits of the respiratory chain complexes of mitochondria, as demonstrated using Western blot analysis of specific proteins (Figure 2e,f): NDUFB8, subunit of Complex I; SDHD, subunits of Complex II; UQCRC2, subunit of Complex III; MTCO1, Complex IV subunit; ATP5A, subunit α of F_O_F_1_-ATPase, Complex V. We demonstrate that in SUM159 cells treated with 150 nM of TR-57 for 24 and 48 h, the expression of subunits of Complexes I-IV decreases gradually as shown in Figure 2e,f. On average, the expression of NDUFB8 decreased by 48% and 86%, SDHD by 65% and 90%, and subunit III UQCRC2 complex by 55% and 77%, respectively. The content of the mitochondrial-encoded subunit of complex IV decreased by 76% already in the first 24 h of incubation with TR-57. Incubation of SUM159 with TR-57 for 72-h and longer resulted in a comparable decline in the expression of the majority of these proteins to ~10% of the initial level of expression (untreated cells), except for ATP5A, Complex V, mitochondrial ATPase α subunit, which decreased by 40% only in the first 24 h exposure to TR-57 (Figure 2f).

Surprisingly, in spite of mitochondrial dysfunction induced by TR57 treatment (inhibition of the respiratory chain activity) under the conditions of our experiments, we were unable to detect any significant changes in the level of ROS monitored using fluorescent probe CM-H2DCFDA.

### 2.3. Effect of TR-57 on Mitochondrial Membrane Potential

**Figure 3:**
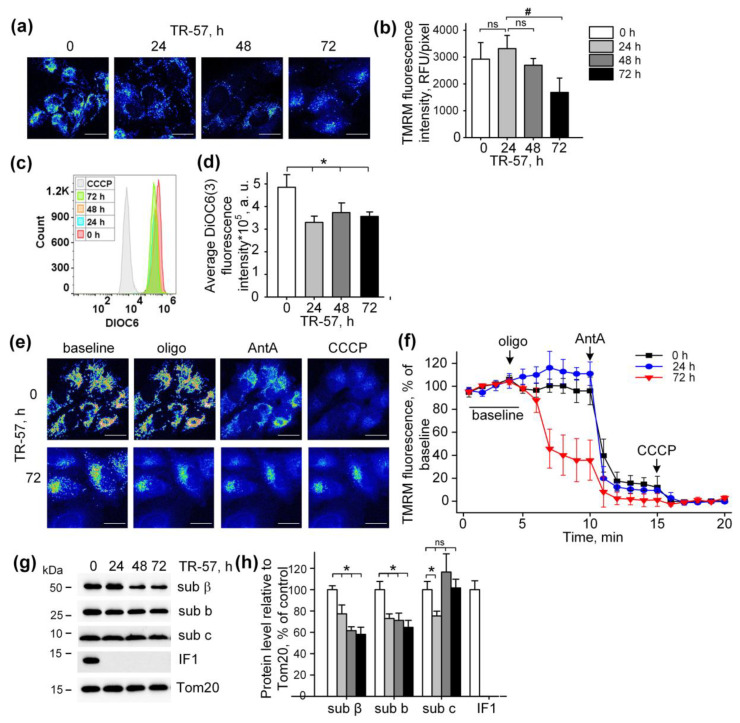
Effect of TR-57 treatment on mitochondrial membrane potential of SUM159 cells. (**a**) Representative confocal fluorescent images of SUM159 cells treated with 150 nM TR-57 for 0 (control), 24, 48 and 72 h and stained with 20 nM TMRM; (**b**) Average intensity of TMRM in mitochondria of SUM159 from panel (**a**); (**c**,**d**) Mitochondrial membrane potential of control (0) and TR-57-treated SUM159 cells measured with DiOC3(6) fluorescent dye by flow cytometry. DiOC3(6) fluorescence intensity of the control cells incubated with 5 µM CCCP for 30 min was taken as positive control; (**e**) Representative confocal fluorescent images of SUM159 cells treated with 150 nM TR-57 for 0 (control) and 72 h, stained with 20 nM TMRM and sequentially exposed to 2 µM Oligomycin (Oligo), 5 µM Antimycin A (AntA) and 1 µM CCCP; (**f**) Relative mitochondrial membrane potential of SUM159 from panel E during sequential addition of Oligo, AntA and CCCP; fluorescence of TMRM was normalized to 100% baseline and 0% after CCCP addition. (**g**,**h**) Representative Western blots and quantitative analysis of F_O_F_1_-ATPase proteins of SUM159 cells following treatment with 150 nM TR-57 for 0 (white bars), 24 h (light gray bars), 48 h (dark gray bars) and 72 h (black bars). Tom20 was used as a loading control. The ratio of protein level to Tom20 under control conditions was taken as 100%. Panels (**a**,**e**) are shown in pseudo color (Fiji LUT—16_colors)The scale bar is 20 µm. The data are presented as the means ± SD of at least three independent experiments. The data were analyzed using a one-way ANOVA with post-hoc Bonferroni test. * *p* > 0.001, # *p* > 0.01, “ns”—non-significant.

The mitochondrial membrane potential is the major component of the electrochemical gradient of a proton across the inner mitochondrial membrane generated by an intact respiratory chain [5]. Despite the complete inhibition of the respiratory chain activity and induction of mitochondrial fragmentation, exposure of cultured SUM159 cells to TR-57 was accompanied by minor mitochondrial depolarization (Figure 3). Mitochondrial membrane potential in cultured SUM159 cells was assessed using two different potential-sensitive fluorescent dyes, TMRM and DiOC6(3), as shown in Figure 3a,b and Figure 3c,d, respectively. Confocal images demonstrate that TMRM fluorescence decreased, but the calculated fluorescence intensity per pixel, which actually reflects the level of potential in individual mitochondria, remained unchanged in the first 48 h of incubation and reduced by approximately 40% after 72 h of incubation (Figure 3a,b). Similarly, using DiOC6(3) and the flow cytometry of the SUM159 cells, we demonstrated a retention of 70% of mitochondrial polarization in these cells after 24, 48 and 72 h of exposure to TR-57 (Figure 3c,d). As shown in the histogram in Figure 3c, the fluorescence intensity of cells incubated with mitochondrial uncoupler CCCP was an order of magnitude lower than that of cells treated with TR-57, which confirms the insignificant effect of TR-57 treatment on the mitochondrial membrane potential.

Typical confocal images of SUM159 cells with TMRM-stained mitochondria subjected to sequential treatment with Oligomycin (inhibitor of F_O_F_1_-ATPase), Antimycin A (inhibitor of the respiratory chain) and CCCP (protonophoric uncoupler of oxidative phosphorylation) demonstrate the level of depolarization of mitochondria after each specific inhibitor in intact and TR-57-treated cells (Figure 3e). Quantifying the intensity of TMRM in intact SUM159 using described protocols demonstrated time-dependent changes in the fluorescence of TMRM induced with Oligomycin, Antimycin A and CCCP (Figure 3f). In control cells, Oligomycin did not cause a noticeable decrease in TMRM fluorescence and, consequently, the potential, while the addition of Antimycin A led to a drop in potential by 80% and the residual membrane potential was depolarized in the presence of CCCP (Figure 3e,f). Applying the same protocol of inhibitors to TR-57-treated SUM159 cells allowed us to monitor the effect of TR-57 exposure on the source of TR-57-insensitive membrane potential. The cells incubated with TR-57 for 24 h demonstrated that the sensitivity of TMRM fluorescence to inhibitors was similar to that in control cells (compare Figure 3f, 0 h, and 24 h). In contrast, cells treated with TR-57 for 72 h demonstrated a 65% decrease in the intensity of TMRM fluorescence after inhibition of F_O_F_1_-ATPase with Oligomycin, and addition of Antimycin A caused the drop of the fluorescence to almost zero level, and the uncoupler had no further effect (Figure 3f, 72 h). In contrast, cells treated with TR-57 for 72 h demonstrated a 65% decrease in the intensity of TMRM fluorescence after inhibition of F_O_F_1_-ATPase with Oligomycin, and addition of Antimycin A caused the drop of the fluorescence to almost zero level, and the uncoupler had no further effect.

Thus, we have shown that in control cells and cells treated for 24 h with TR-57, the mitochondrial membrane potential is supported due to the work of the respiratory chain, while in cells treated with TR-57 for longer time (72 h) the potential is generated mainly due to ATP hydrolysis by mitochondrial F_O_F_1_-ATPase.

Mitochondria are capable of generating inner membrane potential not only through respiration but also through the hydrolysis of ATP to ADP by mitochondrial ATP synthase in reverse mode [27]. In this regard, we studied if TR-57 exposure affects the intactness and composition of proteins of F_O_F_1_-ATPase. As shown in Figure 2e,f, the content of subunit α of F_O_F_1_-ATP synthase in SUM159 cells decreased by 40% within the first 24 h of incubation with TR-57 and remained unchanged with prolonged (72 h) treatment. TR-57 also induced a similar decline in expression of the β protein of the F_1_ catalytic subunit of ATP synthase, located in the matrix, which decreased by 25% after 24 h of incubation and by 40% after 48–72 h of incubation (Figure 3g,h). The level of subunit b of membrane portion F_O_ of ATP synthase decreased by 30–40% within 24 h and remained at this level until 72 h of incubation of cells with TR-57. The level of another subunit of the F_O_, subunit c, which forms the c-ring channel in the inner membrane, through which the H+ ion is transported during ATP synthesis, drops by 20% in the first 24 h of treatment of TR-57 cells, and then even increased by 20% compared to control cells. This increase in protein level may be due to the accumulation of the subunit c in the cell, which occurs under certain pathological conditions and may be a consequence of its reduced degradation [28] and increased expression [29]. However, treatment of SUM159 cells with the TR57 compound resulted in a complete disappearance of IF1, a natural inhibitor of the hydrolysis activity of ATP synthase (Figure 3g,h). Thus, the preservation of a sufficient number of copies of ATPase and the absence of the hydrolysis inhibitory factor IF1 allows us to say that when treated with TR-57, the mitochondria of SUM159 cells can maintain the membrane potential due to the reversal of FoF1-ATPase.

### 2.4. Effect of TR-57 on the Content of Mitochondrial Transport Proteins

**Figure 4:**
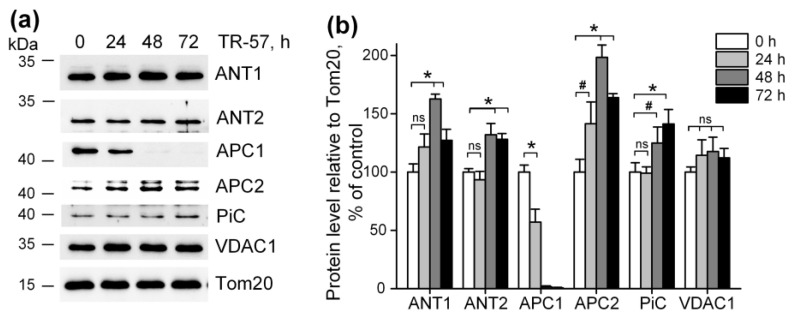
Effect of TR-57 on mitochondrial proteins responsible for ATP and phosphate ion transport. (**a**) Representative Western blots of proteins of control (white bars) SUM159 cells and cells treated with 150 nM TR-57 for 24 h (light gray bars), 48 h (dark gray bars) and 72 h (black bars); (**b**) Quantitative analysis of target protein normalized to Tom20. The ratio of protein level to Tom20 under control conditions was taken as 100%. The data are presented as the means ± SD of at least three independent experiments. The data were analyzed using a one-way ANOVA with a post-hoc Bonferroni test. * *p* > 0.001, # *p* > 0.01, “ns”—non-significant.

It was previously shown that imipridones (ONC201 and/or TR-57) induce the switch of metabolism from oxidative-phosphorylating to glycolytic in many cell cultures [13,14,15,17,30,31], including SUM159 cells. Our observation that mitochondrial dysfunction caused by TR-57 was associated with partial depolarization of mitochondria indicates that mitochondrial membrane potential in TR-57-treated cells is maintained through reversal of mitochondrial F_O_F_1_-ATPase and utilization of the cytosolic ATP, transported into the matrix via transporters. We examined the content of some proteins that may be involved in ATP transport into mitochondria and the dynamics of their changes when cells were treated with TR-57. Figure 4a,b show that the level of voltage-dependent anion channel protein (VDAC1), which transports ATP as well as other nucleotides and ions across the outer mitochondrial membrane, did not change during incubation with TR-57 up to 72 h, and even a slight (non-significant) increase in its content is observed. The level of the proteins ATP/ADP transporters ANT1 and ANT2, responsible for the voltage-dependent exchange of ATP for ADP through the inner mitochondrial membrane, increased by 20% (ANT1) or did not change (ANT2) at 24 h of incubation with TR-57. After 48 h, an increase in protein levels by 60% and 30%, respectively, were observed; by 72 h of treatment with TR-57, the content of ANT1/2 slightly decreased but still remained 20% higher than in control cells. Mitochondria also contain the channels that can transport ATP into the matrix in exchange for inorganic phosphate (Pi)—the so-called calcium-regulated mitochondrial ATP-Mg/Pi carriers (APCs or SCaMCs). As shown in Figure 4a,b, the abundance of APC1 (SCaMC-1, SLC25A24) reduced by 40% after 24 h, and this protein almost completely disappeared after 48 h of incubation with TR-57. However, the expression of another isoform, APC2 (SCaMC-3, SLC25A23), increased by 40% in the first 24 h and doubled after 48 h of incubation. In addition, an increase in the content of inorganic phosphate transport protein (PiC, SLC25A3) is observed up to 40% after 72 h of incubation with the drug. Thus, we can say that ATP transport into the mitochondrial matrix of TR-57-treated cells for subsequent hydrolysis occurs through ANT1/ANT2 or, more likely, with the participation of APC-2.

## 3. Discussion

Mitochondria are key multifunctional intracellular organelles determining the life and death of all cell types through regulation of intracellular supply of ATP, intracellular Ca2+ and redox signaling, metabolic remodeling, apoptotic cell death and intercellular communication [32]. Recent discoveries highlight the role and importance of mitochondria for the initiation and development of a variety of cancers (for review, see [33]). Oncotic diseases such as leukemia, lymphoma, lung adenocarcinoma, pancreatic ductal adenocarcinoma, as well as cancer stem cells with high metastatic and tumorigenic potential, require large amounts of ATP, fulfilled by upregulation of oxidative phosphorylation [34]. In addition to the production of ATP, mitochondria are involved in the catabolic processes of de novo synthesis of nucleotides, lipids and amino acids necessary for proliferating cells, the formation of ROS signaling molecules, calcium signaling pathway and cell death regulation [35]. In addition, targeting specific mitochondrial metabolism is gaining attention as an opportunity for developing efficient cancer cell therapy [35].

Imipridones, a new class of antitumor agents, selectively target mitochondria by activating the unique mitochondrial caseinolytic serine protease ClpP [2,9,10]. It was shown in the in vitro cell culture model that degradation of mitochondrial DNA and morphological changes in mitochondria develop within the first hours (3–6 h) after the start of treatment with the drug [13], but an increase of the level of integrated stress response proteins (ATF-4 and CHOP) occurs at later stages (24–48 h) [2]. Thus, mitochondrial effects are associated both directly with the proteolysis of mitochondrial proteins and indirectly through ClpP-dependent activation of the integrated stress response [2,9,13]. It has been demonstrated that the majority of mitochondrial processes are disrupted by the action of imipridones, such as imipridone-mediated degradation of mtDNA, a number of structural and functional proteins of electron-transport chain, tricarboxylic acid cycle, purine and amino acid metabolism, folate-mediated one-carbon metabolism and proline biosynthesis [9,17,24,31].

In our work, we studied the time-dependent effect of TR-57 on the morphological and functional characteristics of mitochondria in triple-negative breast cancer cells SUM159. Using SUM159, we demonstrated that TR-57 induces mitochondrial fragmentation, inhibition of the activity of the respiratory chain, and decline in the number of mtDNA within the first 24 h of exposure of SUM159 cells to 150 nM of TR57. Longer incubation of these cells with TR57 causes neither a further decrease in mitochondrial size nor a change in mitochondrial mass. At the same time, we observed a decline in the functional state of mitochondria, which was revealed by the inhibition of mitochondrial respiration in a model of digitonin-permeabilized cells, oxidizing substrates of ETC Complexes I and II. A manifestation of a decrease in the rate of oxygen consumption following TR57 exposure occurs within 24 h of treatment. Long-term, 72 h incubation with TR-57, leads to complete inhibition of the respiratory chain of mitochondria in SUM159 cells, as we already observed when studying the effect of ONC201 on BT474 cells [21].

A decrease in the activity of respiratory chain complexes can be due to both inhibition of activity and a decrease in the content of complexes. As was previously shown in glioblastoma cells, lymphoblastic leukemia cell lines and breast cancer cells, substances of the imipridone group and TR-compounds induce a decrease in the levels of enzymes of Complex I and Complex II of the respiratory chain [9,13,14,15,16]. We have shown that during the first 24 h of treatment of SUM159 cells, the protein content of all four complexes of the mitochondrial respiratory chain drops by more than 50%, and after 72 h with the agent, no more than 10% of tested proteins remain. At the same time, the content of F_O_F_1_-ATPase subunits decreased by 40% on the first day and did not change during further incubation, which correlated with changes in mitochondrial mass. This suggests that TR-57 does not affect the distribution of F_O_F_1_-ATPase in mitochondria. According to literature data, one of the subunits of F_O_F_1_-ATP synthase, ATP5H, may be a substrate of the ClpXP complex [36]. However, it is known that imipridones, by binding directly to ClpP, activate it without the participation of ClpX [9,37], so, in this case, the protease substrates may differ from the classical ones. In addition, detailed studies of the proteome and transcriptome of cells (including SUM159) treated with ONC201 or TR-57 [9,10,24] do not indicate up- or downregulation of F_O_F_1_-ATP synthase subunits. A finding of great importance is that ATPIF1, the natural mitochondrial ATPase inhibitor protein, was eliminated early in TR-57 treatment. Previously, proteomic analysis revealed downregulation of IF1 in NALM-6 and SUM 159 cells treated with ONC201 [10,24], which is the opposite of an increased expression of IF1 observed in various types of carcinomas and its role in the metabolic shift from oxidative phosphorylation toward glycolysis has been discussed [38]. It has been shown that IF1 promotes proliferation, migration, and invasion of tumor cells [38], as well as selective knockdown of the IF1 gene in bladder cancer-induced cell proliferation and colony formation [39]. Interestingly, degradation of IF is carried out by an unidentified serine protease [40], and ClpP is precisely this type of protease, allowing one to explain the elimination of IF1 TR-57-induced activation of ClpP and that IF1 could play an important role in the antitumor effect of TR-57.

In our experiments, the exposure of SUM159 cells to imipridone TR-57 resulted in the complete inhibition of mitochondrial respiration and downregulation of key ETC proteins. However, TR-57-treated cells maintained the mitochondrial membrane potential at a relatively high level. In SUM159 cells treated with TR-57 for 24 h and less, with a partial decline in expression of mitochondrial ETC complex proteins and incomplete inhibition of the mitochondrial respiratory chain activity was sufficient to maintain mitochondrial membrane potential. With long-term treatment with TR-57 and associated inhibition of respiration and substantial decline in the expression of the key component of mitochondrial ETC, mitochondrial membrane potential maintained by the reversed activity of F_O_F_1_-ATPase, as suggested by dissipation of mitochondrial potential in the presence of oligomycin, a pharmacological inhibitor of ATPase. The post-TR-57 level of the mitochondrial membrane potential was significantly lower than that maintained by respiration in intact cells, which is in line with the previous observation that the membrane potential generated by ATP hydrolysis is lower than that supported by the respiratory chain alone [41].

We found that exposure of SUM159 cells to TR-57 for up to 72 h did not change the expression of VDAC1, ANT1 and ANT2—the major proteins involved in the transport of ATP into the mitochondria of SUM159 cells. ANTs carry out the electrogenic exchange of ATP for ADP across the membrane; the direction of exchange depends on the concentration of a particular nucleotide on different sides of the membrane [42]. It has been demonstrated that ANT2 in tumor cells transports glycolytically synthesized ATP in exchange for ADP into the mitochondrial matrix and is even considered a marker of proliferation in tumor cells [43]. However, Maldonado et al. showed that neither chemical translocase inhibitors nor genetic knockdown of ANT2/3 in tumor cells affected the level of mitochondrial potential maintained by ATP hydrolysis and concluded that ANT in tumor cells does not participate in the transport of ATP in mitochondria [44]. Under conditions of low mitochondrial membrane potential, as well as when it is necessary to quickly replenish the ATP content in mitochondria, ATP transport can occur through calcium-regulated mitochondrial ATP-Mg/Pi carriers (SCaMC, APC1/APC2), presenting in cells as four paralogues [42].

Some publications show that the APC1 isoform is mainly present in tumor cells [45], and is also upregulated in different types of breast cancer cells [46]. However, in our experiments, we observed a decrease in the level of APC1 during short-term treatment of SUM159 cells with TR-57 and an almost complete disappearance of this protein after 48 h. At the same time, the APC2 isoform increased almost 2-fold at this time point. The content of the inorganic phosphate transporter, which delivers the phosphate necessary for APC function inside the mitochondria, did not change in the presence of TR-57. This allowed us to conclude that the transport of glycolytic ATP into the mitochondrial matrix of TR-57-treated SUM159 cells occurs through APC2. Apparently, despite the fact that the efficiency of ATP transport through APC2 is an order of magnitude lower than through APC1 [47], under the conditions of our work, APC2 isoform alone could be sufficient to maintain the membrane potential in mitochondria with an impaired respiratory chain.

The mitochondrial membrane potential provides a number of functions important for cell life: oxidative phosphorylation, calcium transport into mitochondria, transport of metabolites across the inner mitochondrial membrane, import of mitochondrial proteins, regulation of mitophagy and cell death [48]. In tumor cells, mitochondrial potential determines proliferative and metastatic activity and susceptibility to apoptosis [49]. The question of whether mitochondrial potential is involved in maintaining the viability of TR-57-treated SUM159 cells remains the subject of our further research.

In summary, the effect of the new antitumor agent TR-57 on the mitochondrial population in SUM159 cells resulted in the fragmentation of mitochondria and an essential decrease in the number of mitochondrial nucleoids/mtDNA. Long-term incubation with TR-57 leads to decreased Complexes I–IV protein levels and the complete suppression of mitochondrial respiration. However, even long-term exposure of SUM159 cells to TR-57 does not lead to complete dissipation of the mitochondrial membrane potential. Our preliminary data demonstrate enhanced sensitivity of mitochondrial membrane potential (evaluated using TMRM) toward oligomycin. We speculate that increased levels of ANT-1/2 and APC2 could result in increased transport of cytoplasmic (glycolytic) ATP into the mitochondrial matrix and used by F_O_F_1_-ATPase in reverse mode to support mitochondrial membrane potential in respiratory chain deficient mitochondria. Understanding the mechanism of action of TR-57 on tumor cells opens up new opportunities for its use in pre-clinical and clinical studies in combination with the inhibitors of mitochondrial respiration and membrane potential and/or glycolysis.

## 4. Materials and Methods

### 4.1. Chemicals

TR-57 was provided by Madera Therapeutics, LLC (Chapel Hill, NC, USA). Hoechst 33342, DiOC6(3) and SYBR Green I were obtained from Invitrogen (Waltham, MA, USA), MitoTracker Deep Red 633 and Tetramethylrhodamine methyl ester (TMRM) were from Molecular Probes (Eugenius, OR, USA), DiOC6(3) was from Sigma-Aldrich (St. Louis, MO, USA). DMEM, HEPES and L-glutamine were obtained from PanEco (Moscow, Russia). Fetal bovine serum was from Gibco (Carlsbad, CA, USA).

Antibodies to ATPIF1, APC2 (SLC25A23), TFAM, GAPDH, Tom20 were purchased from Santa Cruz (Santa Cruz, CA, USA), antibodies to subunit β, subunit b, subunit c of mitochondrial ATPase, ANT1, ANT2, APC2 (SLC25A24) and Total OXPHOS antibody cocktail were from Abcam (Cambridge, UK), antibody to TUFM was from Invitrogen (Waltham, MA, USA) and antibody to phosphate carrier (SLC25A23) was from FineTest (Wuhan, China). Secondary goat anti-rabbit antibodies were purchased from PIERCE (Waltham, MA, USA), and secondary horse anti-mouse antibodies were from Cell Signaling (Cell Signaling, Danvers, MA, USA).

Unless otherwise noted, other chemicals used in this work were from Sigma-Aldrich (St. Louis, MO, USA). The concentration of the vehicle (DMSO) used as a solvent for hydrophobic agents was kept under 0.5%.

### 4.2. Cell Culture

Human triple-negative breast cancer cells SUM159 were obtained from the ATCC (Manassas, VA, USA). SUM159 cells were cultured in DMEM/F12 medium supplemented with 5% fetal bovine serum, 2.4 g/L NaHCO_3_, 2 mM L-glutamine, 5 µg/mL insulin, 1 μg/mL hydrocortisone and a 1% mixture of antibiotic-antimycotic in a cell culture incubator (Binder Inc., Bohemia, NY, USA) set at 37 °C in a humidified atmosphere of 5%/CO_2_. SUM159 cells were seeded and cultured overnight in culture dishes (Corning, NY, USA) at 15,000–25,000 cells/sm^2^ density. Following overnight cell adhesion, the incubation media in the experimental dishes was replaced with the fresh media supplemented with 150 nM TR-57, and cells were treated with the drug for 24, 48 and 72 h.

### 4.3. Analysis of Mitochondrial Nucleoids, Mitochondrial Mass and Size

For confocal microscopy experiments, SUM159 cells were plated on 35 mm Petri dishes at a density of 15,000 cells/cm^2^ and treated with 150 nM TR-57 for 24, 48 and 72 h. After treatment, the cells were rinsed three times with 2 mL of HBSS and incubated in 2 mL of HBSS, supplemented with 2 µg/mL Hoechst 33342, SYBR Green I at dilution of 1:200,000, and 150 nM MitoTracker Deep Red 633 at 37 °C for 30 min in CO_2_-free thermostat. Following staining, the cells were washed three times with dye-free HBSS and three-channel fluorescent images of cells were obtained using laser scanning confocal microscope Leica TCS SP-5 DM6000 CS (Leica Microsystems, Mannheim, Germany), at sequential scanning mode using HCX PL APO lambda blue 63x lens, NA = 1.4, Leica Microsystems, Mannheim, Germany). Excitation and emission were set for Hoechst 33342 405 nm/460 nm, SYBR Green I 488 nm/540 nm, and MitoTracker Deep Red 633–633 nm/710 nm. For every sample, five images from random fields were acquired.

Image analysis was performed using Fiji open source software (https://fiji.sc/ (accessed on 1 January 2021)) [25]. We analyzed the number of mt-nucleoids per cell and the average size of mitochondria as described in [26]. Briefly, images were separated into three channels: blue (Hoechst 33342), green (SYBG Green I) and red (MitoTracker Deep Red 633). A mask was created for each channel: mask of nuclei (blue channel), mask of mitochondrial nucleoids (in green channel) and mask of mitochondria (in red channel). The mask from the blue channel was used to quantify the number of nuclei on the image, the green channel was used to quantify the number of mitochondrial nucleoids and the red channel was used to quantify the average mitochondrial size. Additionally, we analyzed the intensity of MitoTracker Deep Red fluorescence as a parameter of mitochondrial mass. For this purpose, we measured the fluorescence intensity of MitoTracker Deep Red within the mask of mitochondria and divided the number of nuclei.

### 4.4. Mitochondrial Respiration in Permeabilized SUM159 Cells

Harvested control and TR-57-treated cells in the amount of 4 × 10^6^ cells per probe were washed once with ice-cold PBS, pelleted at 300× *g* for 2 min and resuspended in 1 mL of respiration medium containing 110 mM KCl, 5 mM NaCl, 5 mM KH_2_PO_4_, 10 mM HEPES (pH 7.4). The incubation medium was supplemented with 5 mM glutamate + 5 mM malate (Complex I substrate) or 10 mM succinate + 1 μg/mL rotenone (Complex II substrate). Cells transferred into 1 mL of the closed measuring chamber of the multichannel recorder FluoFlux-Æ1 (Econix-Expert Inc., Moscow, Russian, webpage: http://ionomer.ru (accessed on 1 December 2023)) and mitochondrial respiration was measured using a dissolved oxygen sensor based on the phosphorescence quenching method [50]. After the permeabilization of the cellular plasma membrane with 0.003% digitonin and subsequent additions of 2 mM ADP, 5 μM carboxyatractyloside (CATR), 30 μM 2,4-dinitrophenol (DNP) and 1 mM NaN3, as described in [21].

### 4.5. Measurement of Mitochondrial Membrane Potential and Mitochondrial Mass Using Flow Cytometry

The intact and TR-57-treated SUM159 cells were washed three times with PBS and detached from the surface of the culture plastic using a 0.05% trypsin-EDTA solution. Cell aliquots containing equal cell amount (500,000 cells) were stained for 30 min in a CO_2_ incubator in the full incubation medium (DMEM/F12) with 10 nM 3,3′-dihexyloxacarbocyanine iodide (DiOC6(3)) to measure the mitochondrial membrane potential or with 50 nM MitoTracker Deep Red to assess mitochondrial mass. Experimental data were obtained using a BD Accuri C6 flow cytometer (BD Bioscience, Franklin Lakes, NJ, USA) were processed using BD Accuri C6 CFlow software 1.0.264.21 (BD Bioscience, Franklin Lakes, NJ, USA) and resulting histograms were plotted using FlowJo v10 software (BD Bioscience, Franklin Lakes, NJ, USA).

### 4.6. Membrane Potential Measurement in Intact Cells Using Fluorescence Microscopy

SUM159 cells were plated on 0.13 mm thick 25-mm round coverslips (Menzel-Gläser, Braunschweig, Germany) in 35 mm Petri dishes at a density of 10,000 cells/cm^2^ and treated with 150 nM TR-57 for 24, 48 and 72 h. Following TR-57 treatment, coverslips with cells were incubated in 1 mL of complete incubation medium containing 20 mM HEPES (PanEco) and 20 nM TMRM for 30 min in a cell culture incubator (95/5% air/CO_2_). After staining, the incubation media was replaced with a similar one containing 20 nM TMRM, and the cells were transferred to a microscopic stage. A Leica DMI6000 B fluorescence microscope (Leica microsystems, Mannheim, Germany) equipped with Hamamatsu EM-CCD digital camera C9100 (Hamamatsu Photonics, Hamamatsu, Japan), the OSRAM HXP R 120W/45C UV mercury lamp, HC PL APO 20x/0.70 water immersion lens, green excitation filter (530 nm), red 640 mm emission filter were used to evaluate TMRM fluorescence. Exposure time was set to 100 ms. 2 × 2 camera binning, gain 1100, an additional magnification lens 1.6x and 50% neutral density filter were used to decrease excitation light intensity. Time-lapse images were acquired frame by frame with an interval of 1 frame per minute. The baseline (initial TMRM fluorescence) was taken for 5 min, then 2 μM oligomycin was added to the cells, and the change in TMRM fluorescence was recorded for 5 min, after that 5 μM antimycin A and 1 μM CCCP uncoupler were added, and the images were acquired for 5 min each. The obtained stacks were analyzed using Fiji open source software (https://fiji.sc/ (accessed on 1 January 2021)) as described [25]. The level of mitochondrial membrane potential was determined as the intensity of TMRM fluorescence averaged from 5 min of baseline. For tracking of changes in mitochondrial membrane potential in each series, 10 regions of cell mitochondria and 10 corresponding regions of nuclei and 1 region of the extracellular environment were selected, and the fluorescence of TMRM was measured. The relative level of membrane potential (Δ*ψ*) was calculated using the following formula:

Δ*ψ* = (F_mt_ − F_background_)/(F_nucleus_ − F_background_)

where F_mt_ is the average fluorescence level of the mitochondrial region of the cell, F_nucleus_ is the average fluorescence level of the cell nucleus area, and F_background_ is the average fluorescence level of the extracellular area. The obtained values of membrane potential at each point were normalized to baseline level (referred to as 100%) and CCCP (referred to as 0%).

### 4.7. Western Blot Analysis of Mitochondrial Proteins

Intact and TR-57-treated cells (4 × 10^6^) were trypsinized, washed twice in PBS, pelleted at 300× *g* for 5 min and lysed in RIPA Lysing Buffer System with 1 mM Na_3_VO_4_, 2 mM PMSF, and a complete protease inhibitor cocktail (Santa Cruz, CA, USA). Samples were solubilized in Laemmli loading buffer (BioRad, Hercules, CA, USA) and boiled at 95 °C for 5 min (except samples for Total OXPHOS detection heated at 37 °C for 5 min). The concentration of protein in samples was measured by the Bradford method, and 30 μg of each sample was subjected to PAAG electrophoresis followed by transfer to nitrocellulose membrane. Then, membranes were blocked with 5% dry milk in PBST for 1 h at room temperature and incubated overnight at 4 °C with primary antibodies against target proteins. After incubation with corresponding secondary antibodies conjugated with HRP for 1h at room temperature, target proteins were visualized with ClarityWestern ECL substrate (Bio-Rad, Hercules, CA, USA). Chemiluminescence was detected with Chemidoc Touch Imaging System (Bio-Rad, Hercules, CA, USA) and ImageLab 5.2 software was used for the processing and quantification of the obtained results.

### 4.8. Statistical Analysis

All experiments were performed at least in triplicate and were analyzed using the ANOVA one-way variance analysis with a post-hoc Bonferroni test [21]. The values are presented as mean ± SD and statistical significance of *p* < 0.05.

## Data Availability

Data are available within the article and Supplementary Materials.

## Supplementary Materials

The following supporting information can be downloaded at: https://www.mdpi.com/article/10.3390/ijms25021193/s1.
