# Supplementary material for: TR-57 Treatment of SUM159 Cells Induces Mitochondrial Dysfunction without Affecting Membrane Potential
Source: Int J Mol Sci. 2024 Jan 18;25(2):1193. doi: 10.3390/ijms25021193 (PMC10816083; doi:10.3390/ijms25021193)
Supplement: Supplementary file 1 [file ijms-25-01193-s001.zip › ijms-2782534-SI.pdf]

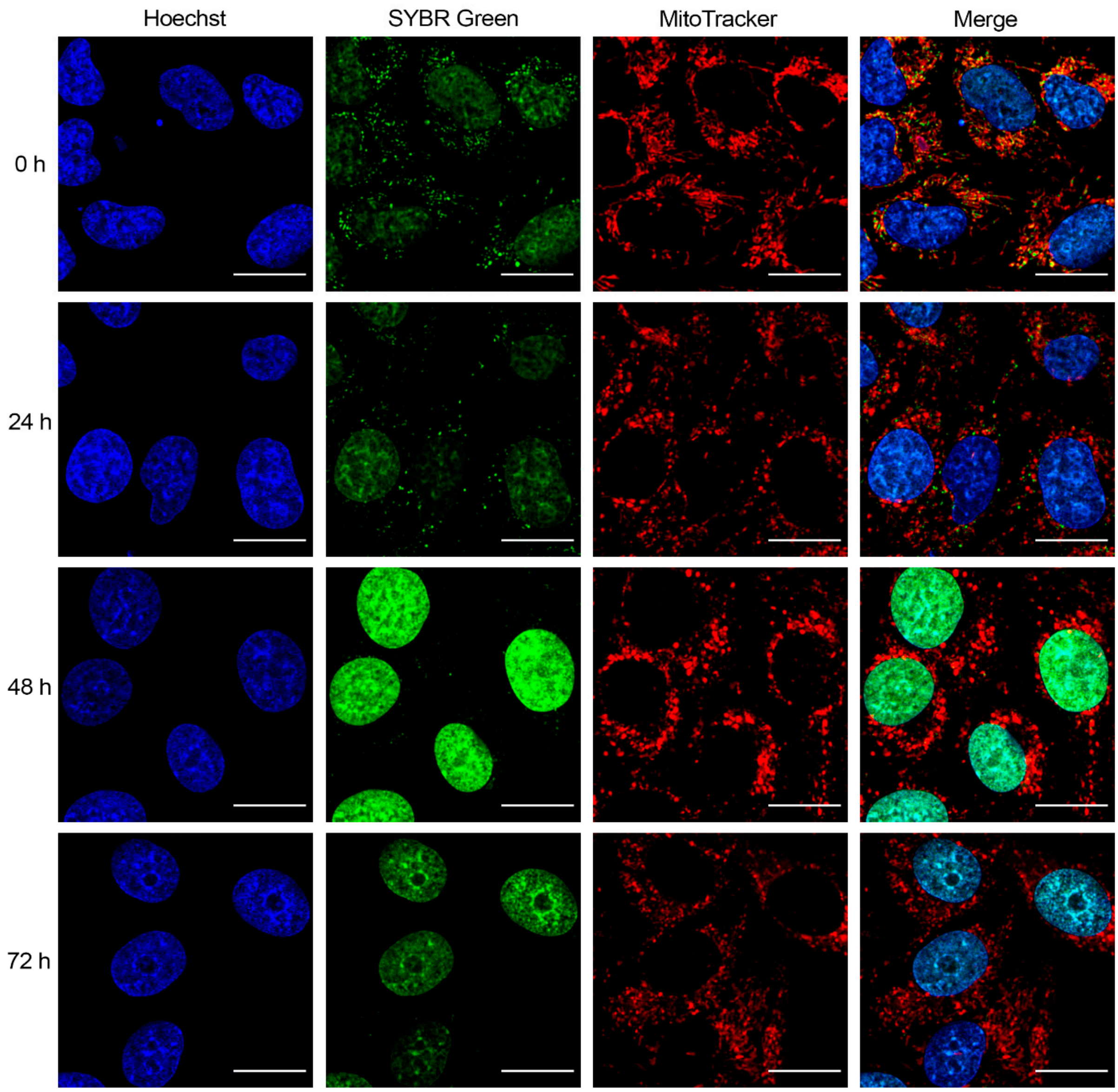

Figure S1. Representative confocal fluorescent images of intact (0 h) and treated with 150 nM TR-57 for 24, 48 and 72 hours SUM159 cells loaded with Hoechst 33342 (Hoechst, blue), SYBR Green I (SYBR, green) and MitoTracker Deep Red (MTDR, red); The scale bar is 20 μm.
